# Twenty-four-week oral dosing toxicities of Herba Siegesbeckiae in rats

**DOI:** 10.1186/s12906-020-03137-6

**Published:** 2020-11-11

**Authors:** Jia-Ying Wu, Yuen-Cheung Chan, Hui Guo, Ying-Jie Chen, Yu-Xi Liu, Hua Yi, Zhi-Ling Yu

**Affiliations:** 1 Research and Development Centre for Natural Health Products, HKBU Shenzhen Research and Continuing Education, Shenzhen, China; 2 Centre for Cancer and Inflammation Research, School of Chinese Medicine, Hong Kong Baptist University, Kowloon Tong, 999077 Hong Kong; 3 Department of Pathology, Guangzhou University of Chinese Medicine, Guangzhou, China

**Keywords:** Herba Siegesbeckiae, Hepatotoxicity, Pulmonary toxicity, Chronic toxicity, Rats

## Abstract

**Background:**

Herba Siegesbeckiae (HS), the dried aerial parts of *Siegesbeckia orientalis* L., *S. pubescens* Makino, or *S. glabrescens* Makino, is traditionally used for treating chronic diseases in China. However, there is no information about the chronic toxicity of HS. The objective of this study is to evaluate the 24-week oral dosing toxicities of HS aqueous extract (HSE) in rats.

**Methods:**

*S. orientalis*-originated HS was reflux-extracted with distilled water. Sprague–Dawley rats were randomly divided into four groups, with 10 males and 10 females in each group. The rats were intragastrically administered with HSE at 5, 1.67 and 0.56 g/kg (experimental groups) or an equal volume of distilled water (control group), 6 days a week, for 24 weeks. The high dose of HSE (5 g/kg) was its maximum tolerated dose. Body weight was recorded every 2 days during the experimental period. Chemical, hematological and histopathological parameters, as well as organ weights, were measured at the end of the experiment.

**Results:**

Decreased body weight gain; increased liver and lung relative weights; histopathological alterations in liver and lung tissues; elevated serum levels of alkaline phosphatase, aspartate aminotransferase, alanine aminotransferase and lactate dehydrogenase were found after HSE treatments. In liver tissues, HSE treatment upregulated levels of three pro-inflammatory cytokines: IL-6, IL-1β and TNF-α. In lung tissues, HSE treatment caused oxidative stress and activated mitogen-activated protein kinases (MAPKs).

**Conclusion:**

Long-term oral administration of HSE caused toxicities in rats evidenced by decreased body weight gain, as well as liver and lung damage. Treatment-induced oxidative stress, inflammation and MAPK activation are involved in HSE’s toxicities. Caution should be taken when using HS to treat chronic diseases.

## Background

Herba Siegesbeckiae (HS), the dried aerial parts of *Siegesbeckia orientalis* L., *S. pubescens* Makino, or *S. glabrescens* Makino, was first recorded as a low-toxicity herb in “*Xin Xiu Ben Cao*” issued in 659 A.D. (Tang Dynasty of China) [1]. This herb, alone or in combination with other herbs, is commonly used for managing chronic diseases such as rheumatoid arthritis (RA) and hypertension by traditional Chinese medicine (TCM) doctors [2]. Chemical studies have demonstrated that *S. orientalis* mainly contains diterpenoids and glucosides; that *S. pubescens* mainly contains organic acids and long-chain alkanols; and that *S. glabrescens* mainly contains long-chain alkanol acids [3]. Bioactive components of HS include kirenol, darutogenol, isodarutogenol, stigmasterol [4]. Pharmacological studies have showed that HS has diverse bioactivities such as anti-bacterial [5], anti-inflammatory [6] and anticancer properties [7, 8].

Despite extensive clinical use of HS, only a few studies about the acute and sub-chronic toxicities of this herb have been conducted. One acute toxicity study showed that the mouse median lethal dose (LD_50_) of the water extract of HS given by intragastrical (i.g.) administration is 18.02 g/kg (equivalent to crude herb 146.7 g/kg) [9]. A sub-chronic toxicity study demonstrated that lung injury occurred in mice i.g. dosed with aqueous extract of HS at a dose of 3.0 g/kg for 2 weeks [10]. Jiang et al. found that HS water extract exerted stronger acute toxicity than its ethanol extract in mice [11]. Given that HS is commonly used for several months or even several years to treat chronic diseases [3, 12] and its chronic toxicity profile is not available, chronic toxicity evaluation of this herb is an urgent issue.

Sprague–Dawley (SD) rats have been widely used to study toxicities of medicinal herbs [13]. In the present study, in order to provide guidance for the clinical use of HS, we tested the 24-week oral dosing toxicities of HS in SD rats.

## Methods

### Herbal materials

A study found that there was no significant difference in acute toxicity among HS samples originating from its three different species [14]. We chose the most commonly used species *S. orientalis* for experiments in this study. HS was purchased from Hubei Shen Nong Traditional Chinese Medicine Co. Ltd. (Hubei, China). The herb was authenticated by Prof. Chen Hubiao, School of Chinese Medicine, Hong Kong Baptist University. Voucher specimen (No. 110904771) was deposited at the School of the Chinese Medicine, Hong Kong Baptist University. Because water extract of HS is more toxic than its ethanol extract, and HS is typically administered in a water decoction [11], we prepared HS as a water extract for investigations in the present study. HS was macerated for 1 h in distilled water (1:10, w/v) at room temperature (25 ± 2 °C), and then reflux-extracted twice at 100 °C, 2 h each time. The combined extracts were evaporated to 1/10 of its original volume under reduced pressure, using a rotary evaporator. Following the “Delta 20 Rule” (10/30/50 parameters), water bath temperature was set as 50 °C and cooling water temperature was set at 10 °C [15]. The vacuum was set as 42 mbar to adjust the boiling point of water to 30 °C [16]. The concentrate was freeze-dried with a Virtis Freeze Dryer to obtain HS extract (HSE for short; yield: 17.05%). HSE was stored at 4 °C. Immediately before use, HSE was dissolved in distilled water.

To control the quality of HSE, a high-performance liquid chromatography (HPLC) analysis was conducted using an Agilent HP 1260 system equipped with a Diode-array detector (Hewlett Packard, Agilent, USA). Separations were performed on an Alltima™ C-18 analytical column (250 mm × 4.6 mm I.D., 5 μm) and an Alltima C-18 guard column (12.5 mm × 4.6 mm I.D., 5 μm). Isocratic elution was performed with a mobile phase of A (0.1% phosphate acid solution, analytical grade, RCI Labscan Limited) and B (ACN, HPLC grade, RCI Labscan Limited) (70:30, v/v). The flow rate was maintained at 0.35 mL/min, and the column temperature was set at 25 °C. Sample injection volume was 5 μL in each test. Since kirenol (marker compound of HS in Chinese Pharmacopoeia) has a prominent absorption around 215 nm in the UV spectrum, 215 nm was chosen as the reference wavelength. The HPLC chromatogram showed that kirenol, an anti-inflammatory compound, is present in HSE (Figure S1a). The content of kirenol in HSE is 162 μg/g (Figure S1b).

### Animals

Male and female SD rats were purchased from the Laboratory Animal Services Centre, Chinese University of Hong Kong. All care and handling of animals were performed with the approval of the Department of Health, Hong Kong. Experimental procedures were approved by the Committee on the Use of Human & Animal Subjects of the Hong Kong Baptist University (HASC/12–13/0023). Rats were housed in plastic cages in our school’s animal facility (25 ± 2 °C, humidity: 60 ± 10%, 12 h-light: 12 h-dark) with free access to water and standard rodent pellet. They were acclimatized for 1 week before being used in experiments.

### Twenty-four-week oral dosing toxicity assessment

In chronic toxicity assessments, the high dose used in animals should allow identifying the principal target organs and toxic effects, while not cause suffering, severe toxicity, morbidity, or death [17]. To determine the high dose of HSE for the 24-week oral dosing toxicity assessment, we tested its acute toxicity first.

In acute oral dosing toxicity assessment, 20 SD rats (170 ± 10 g) were randomly divided into two groups: control group and HSE group, each group comprising 5 males and 5 females. The maximum concentration of HSE in distilled water was 0.34 g/ml, and the maximum dosing volume was 2.5 mL for each rat, the maximum tolerated dose (MTD) of HSE was calculated as 5 g/kg (0.34 g/mL × 2.5 mL ÷ 170 g). One i.g. dosing of HSE at 5 g/kg did not cause any toxic sign or mortality in the 10 rats during a 14-day observation period. Body weights of rats treated with 5 g/kg of HSE or an equal volume of distilled water are shown in Figure S2. In the chronic toxicity assessment of HSE, its MTD (5 g/kg) was used as the high dose. The middle and low doses were 1/3 and 1/9 of the high dose, respectively.

In the 24-week oral dosing toxicity assessment, 80 SD rats (100 ± 15 g) were randomly divided into four groups: control group, high-dose HSE group, medium-dose HSE group and low-dose HSE group; with 10 males and 10 females in each group. Rats in experimental groups were i.g. administered with HSE at the doses of 5, 1.67 and 0.56 g/kg (equivalent to 29.4, 9.8, and 3.3 g/kg crude herb, respectively). Rats in the control group were i.g. dosed with an equal volume of distilled water. All rats were dosed 6 days/week [18] for 24 weeks. During the experimental period, body weight and food intake were recorded every 2 days; and general clinical observations were conducted once daily. The observations included: changes in skin, fur, eyes, secretions, excretions, autonomic activities, gait, posture and response to handling. Rat ophthalmological examinations were carried out prior to the first drug dosing and at the termination of the study. After last dosing, all rats were fasted for 18 h and then anesthetized with an intraperitoneal injection of ketamine (Ketamidor, Richter Pharma AG, Wels, Austria)/xylazine (Xylased, Bioveta, Ivanovice na Hané, Czech Republic) anesthetizing cocktail (50 mg/kg of ketamine and 5 mg/kg xylazine). Blood was collected from the abdominal aorta for further evaluations. After blood collection, animals were immediately sacrificed by cervical dislocation and subjected to postmortem examinations. At necropsy, all organs and tissues were examined for grossly visible lesions.

### Organ weight and histopathology

After rats were sacrificed at the end of the experiment, organs (heart, liver, spleen, lung, kidney, stomach, small and large intestines, brain, adrenal gland, ovary, uterus, testes and epididymides) were quickly removed, and cleared from adipose and connective tissues. All organs were weighed, and organ indexes were calculated. Organ index = organ weight (g)/body weight (g) × 100%.

Following necropsy, tissues used for histopathological analyses were fixed in 10% neutral-buffered formalin overnight at 4 °C, dehydrated with varying grades of alcohol, embedded in paraffin and then sectioned with a rotary microtome. All samples were sectioned at the thickness of 5 μm and stained with hematoxylin (Sigma MHS-16) and eosin (Sigma HT110–1-32) (H&E). Cryosection imaging was performed using an AxioImager Z.1 microscope (Zeiss) with Volocity software.

### Urinalysis

During the last day of the experiment, urine samples were collected, and urine total volumes were recorded. Urine samples were immediately centrifuged at 15,700 g for 10 min. Specific gravity, pH, leukocyte, nitrite, protein, glucose, ketone bodies, urobilinogen, bilirubin and erythrocyte were analyzed using a urine chemistry analyzer (Urisys, Roche, Switzerland) following the manufacturer’s protocols.

### Hematology and serum chemistry

Collected blood samples were examined by Optimal Medical Laboratory Limited Company (Hong Kong) for routine hematological parameters including total leukocyte, differential leukocyte, platelet, haemoglobin, haematocrit, and blood clotting time. For biochemical analyses, blood samples were coagulated at room temperature, and then centrifuged at 3000 g for 10 min. Serum was separated and stored at − 80 °C for further evaluations. Serum levels of sodium (Na^+^), potassium (K^+^), calcium (Ca^2+^), creatine kinase (CK), alkaline phosphatase (ALP), blood urea nitrogen (BUN), creatinine (CRE), total protein (TP), aspartate aminotransferase (AST), alanine aminotransferase (ALT), lactate dehydrogenase (LDH), albumin (ALB), blood glucose (GLU), total cholesterol (TC), and total bile acid (TBA) were analyzed using biochemical reagents purchased from Thermo Fisher Scientific (USA) and Nanjing Jiancheng Bioengineering Institute (China) following manufacturers’ protocols.

### Western blot analysis

Lysates were prepared from rat lung and liver tissues. Each sample was homogenized vigorously with 1 mL of RIPA lysis buffer [50 mM Tris-HCl, 1% NP-40, 0.35% sodium-deoxycholate, 150 mM NaCl, 1 mM EDTA (pH 7.4), 1 mM phenylmethylsulfonyl fluoride, 1 mM NaF, 1 mM Na3VO4 and 10 μg/mL each of aprotinin, leupetin and pepstatin A]. After incubation on ice for 15 min, the homogenate was centrifuged at 14,000 g for 30 min at 4 °C. After that, the supernatants were transferred into new tubes and stored at − 80 °C. Protein concentrations were measured using Quick Start™ Bradford Protein Assay (Bio-Rad, USA).

Western blot assays were performed as described previously [19]. Immunoreactive bands were visualized using the Enhanced Chemiluminescence (ECL) detection system (Invitrogen, Carlsbad, CA, USA). Grey value of each band was measured using Image J software. Relative level of a protein was normalized to the endogenous control α-Actinin in each experiment. Jun N-terminal kinase (JNK, 9252S), phospho-JNK (Thr183/Tyr185, 4668P), p38 (9212S), phospho-p38 (Thr180/Tyr182, 9211S), extracellular signal regulated kinase (ERK, 9102S), phospho-ERK (Thr202/Tyr204, 9101S), α-Actinin (6487S) monoclonal antibodies were purchased from Cell Signaling Technology (Boston, MA, USA). HRP-conjugated secondary antibodies (ab7090, ab97040) were obtained from Abcam (Cambridge, CB2 0AX, UK).

### Enzyme-linked immunosorbent assay (ELISA)

Lysates were prepared from rat liver and lung tissues. Each liver or lung sample was homogenized vigorously with 1 mL of PBS. After incubation on ice for 15 min, the homogenate was centrifuged at 5000 g for 10 min at 4 °C. After centrifugation, the supernatants were transferred into new tubes and stored at − 80 °C. Levels of interleukin (IL)-6, IL-1β and tumor necrosis factor (TNF)-α were measured using respective ELISA kits (R&D Systems, Inc., Canada) following the manufacturer’s protocols.

### Measurements of lipid peroxidation and antioxidant enzyme activities

Lysates were prepared as described in the ELISA section. Malondialdehyde (MDA), glutathione peroxidase (GSH-PX), catalase (CAT) and superoxide dismutase (SOD) of the liver or lung tissue homogenates were measured by colorimetric method using respective kits (Nanjing Jiancheng Biology, Nanjing, China) following the manufacturer’s protocols.

### Statistical analysis

Data are presented as the means ± standard error of the mean (SEM), and were analyzed by one-way analysis of variance (ANOVA) followed by Dunnett’s multiple comparisons using the statistical software GraphPad Prism 6.0 (GraphPad Software, San Diego, CA, USA). *P* < 0.05 was considered statistically significant.

## Results

### HSE decreased the body weight gain of rats

**Fig. 1:**
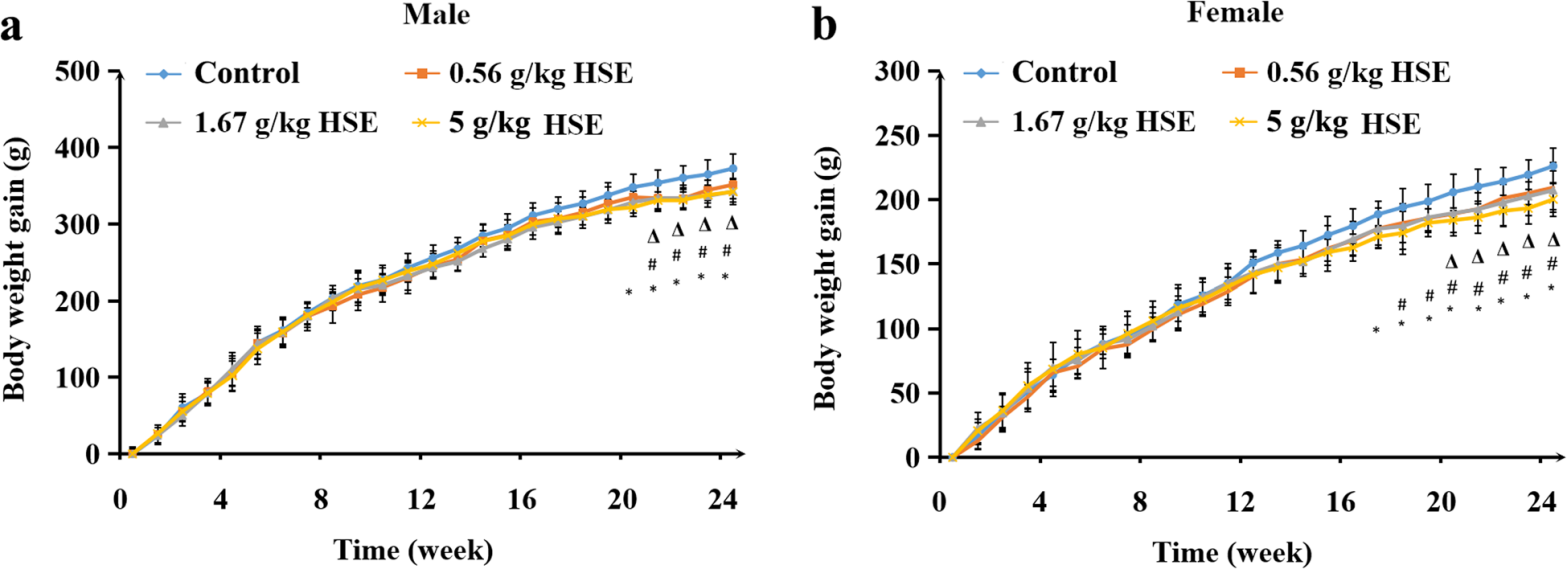
HSE decreased the body weight gain of rats. Rats were randomly divided into four groups: control group, 5 g/kg of HSE group, 1.67 g/kg of HSE group and 0.56 g/kg of HSE group, each 10 males and 10 females. Rats were intragastrically administered with distilled water (control group) or indicated doses of HSE, 6 days a week, for 24 weeks. **a** Body weight gain of male rats. **b** Body weight gain of female rats. Values are expressed as mean ± SEM (*n* = 10). * *P* < 0.05: 5 g/kg HSE group vs. control group. # *P* < 0.05: 1.67 of g/kg HSE group vs. control group. Δ *P* < 0.05: 0.56 g/kg of HSE group vs. control group

During the 24-week experiment period, no rat died, and no obvious abnormality was observed in any rat’s physical appearance or behavior, nor in any rat’s ophthalmological examinations (data not shown). The food intake of rats was not significantly affected by HSE treatments (data not shown). However, dosing with HSE decreased body weight gain (Fig. 1a-b). Male rats treated with 5 g/kg of HSE gained less weight from weeks 20–24; and female rats treated with 5 g/kg of HSE gained less weight from weeks 17–24 than control rats. Male rats treated with 1.67 g/kg of HSE gained less weight from weeks 21–24; and female rats treated with 1.67 g/kg of HSE gained less weight from weeks 18–24 than control rats. Male rats treated with 0.56 g/kg of HSE gained less weight from weeks 21–24; and female rats treated with 0.56 g/kg of HSE gained less weight from weeks 20–24 than control rats. These findings indicate that long-term treatment of HSE decreases body weight gain of rats.

### HSE elevated relative liver and lung weights, and induced histopathological alterations in liver and lung tissues

**Fig. 2:**
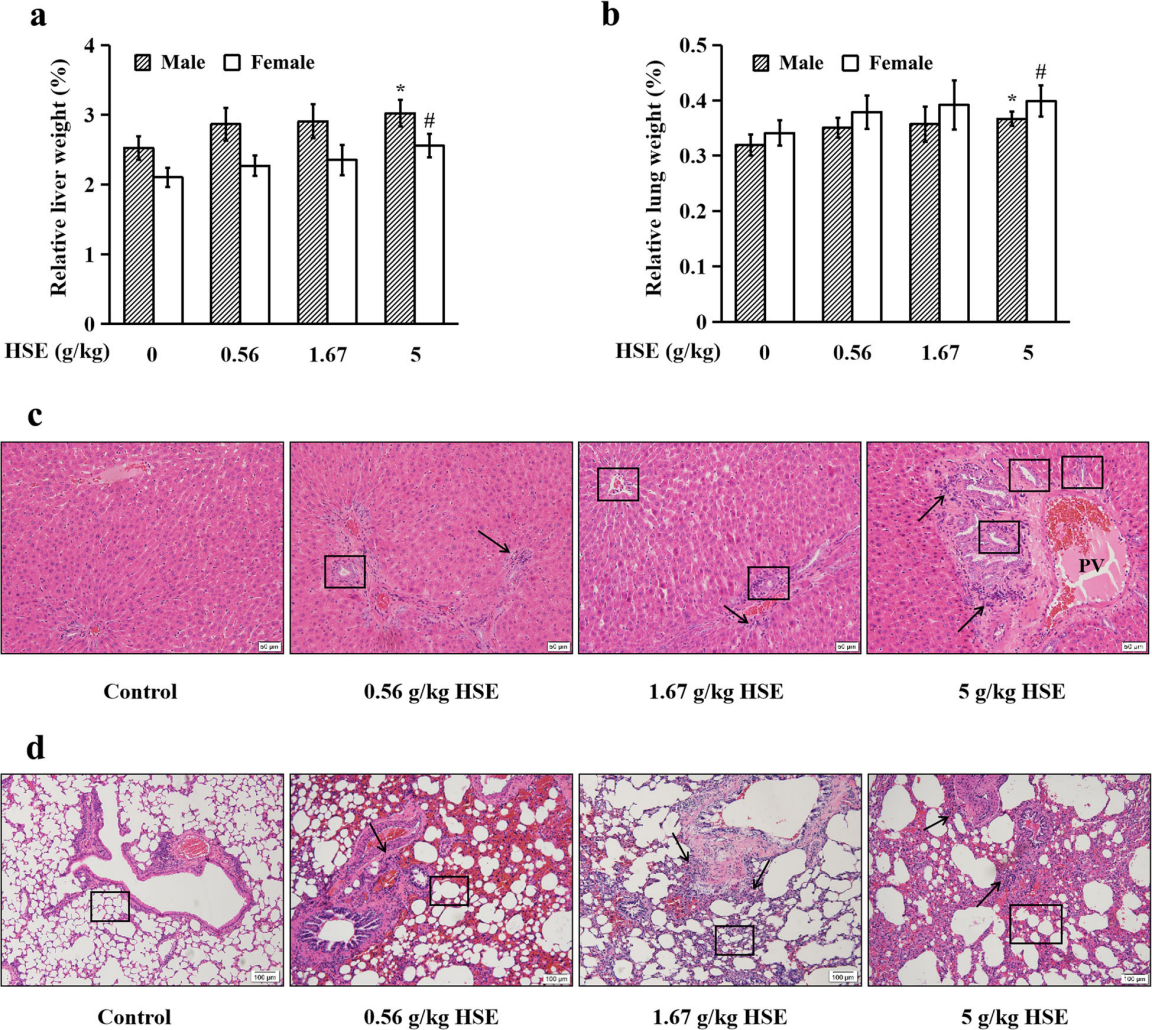
HSE elevated relative liver and lung weights, and induced histopathological alterations in liver and lung tissues. Animal treatments are the same as in Fig. 1. **a** Relative liver weight of rats. **b** Relative lung weight of rats. Values are expressed as mean ± SEM (*n* = 10). * *P* < 0.05 vs. control group of male rats. # *P* < 0.05 vs. control group of female rats. **c** H&E staining of liver tissues. Representative photos of H&E staining of liver tissues are shown. Inflammatory cells (arrows), bile duct (squares), and portal vein (PV) are pointed. Scale bars: 50 μm. **d** H&E staining of lung tissues. Representative photos of H&E staining of lung tissues are shown. Inflammatory cells (arrows) and alveoli (squares) are pointed. Scale bars: 100 μm

The absolute and relative organ weights of rats are shown in Table S1. HSE at 5 g/kg, but not at 1.67 g/kg or 0.56 g/kg, significantly elevated relative weights of liver (Fig. 2a) and lung (Fig. 2b) in rats. The absolute liver and lung weights in all HSE-treated groups were also increased although there was no significant difference compared with the control group. Absolute and relative weights of heart, spleen, kidney, brain, adrenal gland, testis/ovary and epididymis/uterus in all HSE-treated groups were not significantly altered compared with the control group. In all HSE-treated groups, bile duct hyperplasia, and enlarged portal vein were observed in liver tissues (Fig. 2c); and abnormal enlargement of the alveolar spaces, and thickened alveolar walls were found in lung tissues (Fig. 2d). Infiltrations of macrophages and lymphocytes were observed in both liver (Fig. 2c) and lung (Fig. 2d). Histopathological examinations showed no significant difference in heart, spleen, kidney, stomach, small and large intestines, brain, adrenal gland, testis/ovary, epididymis/uterus, thighbone, bone marrow, skeletal muscle, stomach, duodenum, jejunum and colon in all HSE-treated groups compared with the control group (data not shown). These results indicate that chronic treatment with HSE, even at relatively low doses, induces histopathological alterations in rat liver and lung.

### HSE elevated serum levels of ALP, AST, ALT and LDH, and up-regulated levels of pro-inflammatory cytokines in liver tissues of rats

**Fig. 3:**
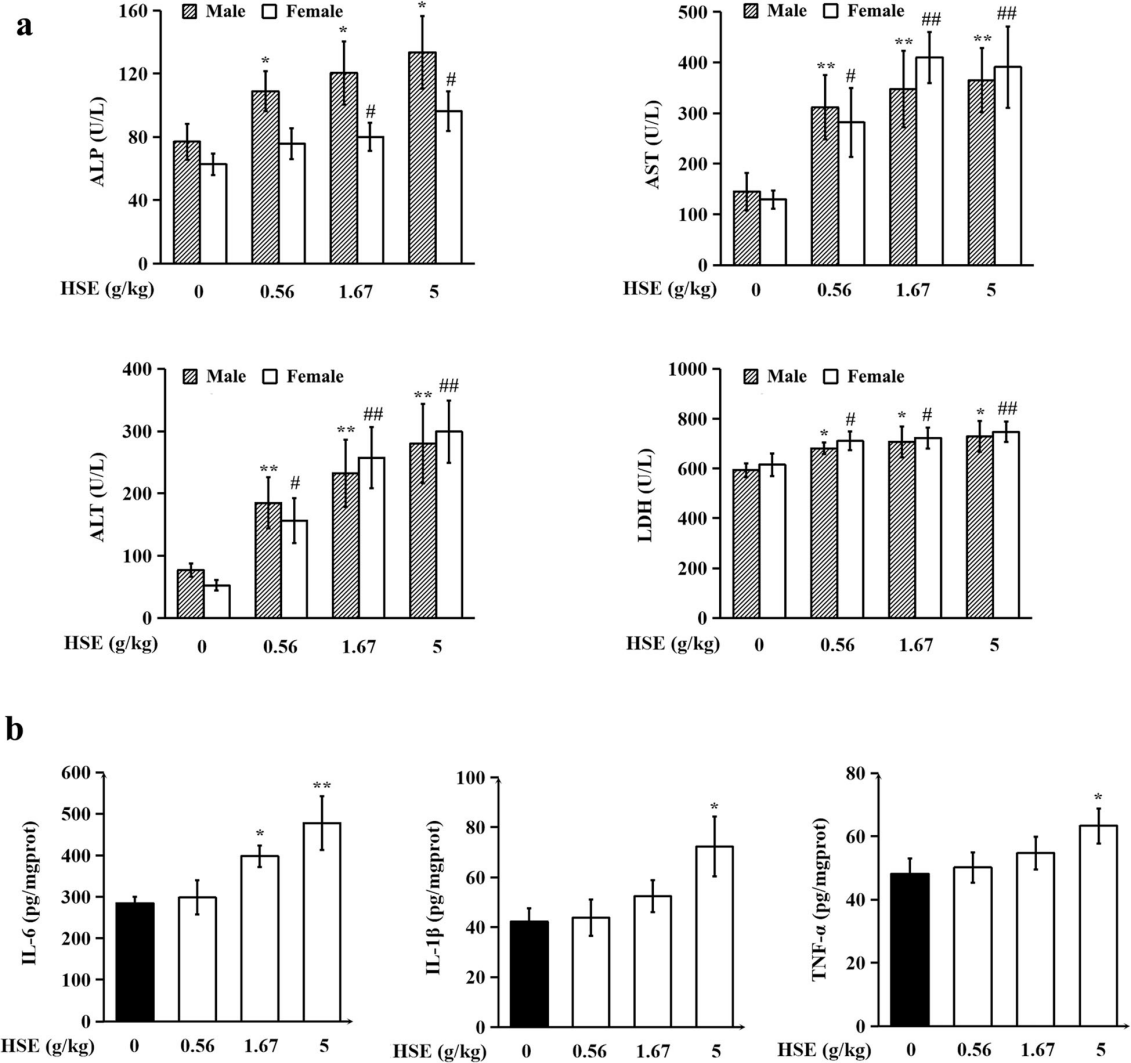
HSE elevated serum levels of ALP, AST, ALT and LDH, and upregulated levels of pro-inflammatory cytokines in liver tissues of rats. Animal treatments are the same as in Fig. 1. **a** Serum levels of ALP, AST, ALT and LDH. Values are expressed as mean ± SEM (*n* = 10). * *P* < 0.05, ** *P* < 0.01 vs. control group of male rats. # *P* < 0.05, ## *P* < 0.01 vs. control group of female rats. **b** Levels of IL-6, IL-1β and TNF-α in liver tissues. Detected using ELISA. Values are expressed as mean ± SEM (*n* = 20). * *P* < 0.05, ** *P* < 0.01 vs. control group

At the end of the experiment, urine, blood and serum samples were collected for detection. As shown in Tables S2 and S3, no treatment-related change was observed in any of the urine parameters or hematological parameters. Figure 3a showed that HSE treatment markedly elevated serum levels of four liver injury biomarkers: ALP, AST, ALT, and LDH, but did not affect serum levels of Na^+^, K^+^, Ca^2+^, CK, BUN, CRE, TP, ALB, GLU, TC, and TBA (Table S4). As infiltration of immune cells was observed in liver tissues of HSE-treated rats, we then detected the levels of pro-inflammatory cytokines in liver tissues. As shown in Fig. 3b, HSE treatments dose-dependently up-regulated protein levels of IL-6, IL-1β and TNF-α. These results demonstrate that chronic treatment with HSE causes liver damage and induces over-production of pro-inflammatory cytokines in liver tissues.

### HSE caused oxidative stress, and induced the activation of mitogen-activated protein kinases (MAPKs) in lung tissues of rats

**Fig. 4:**
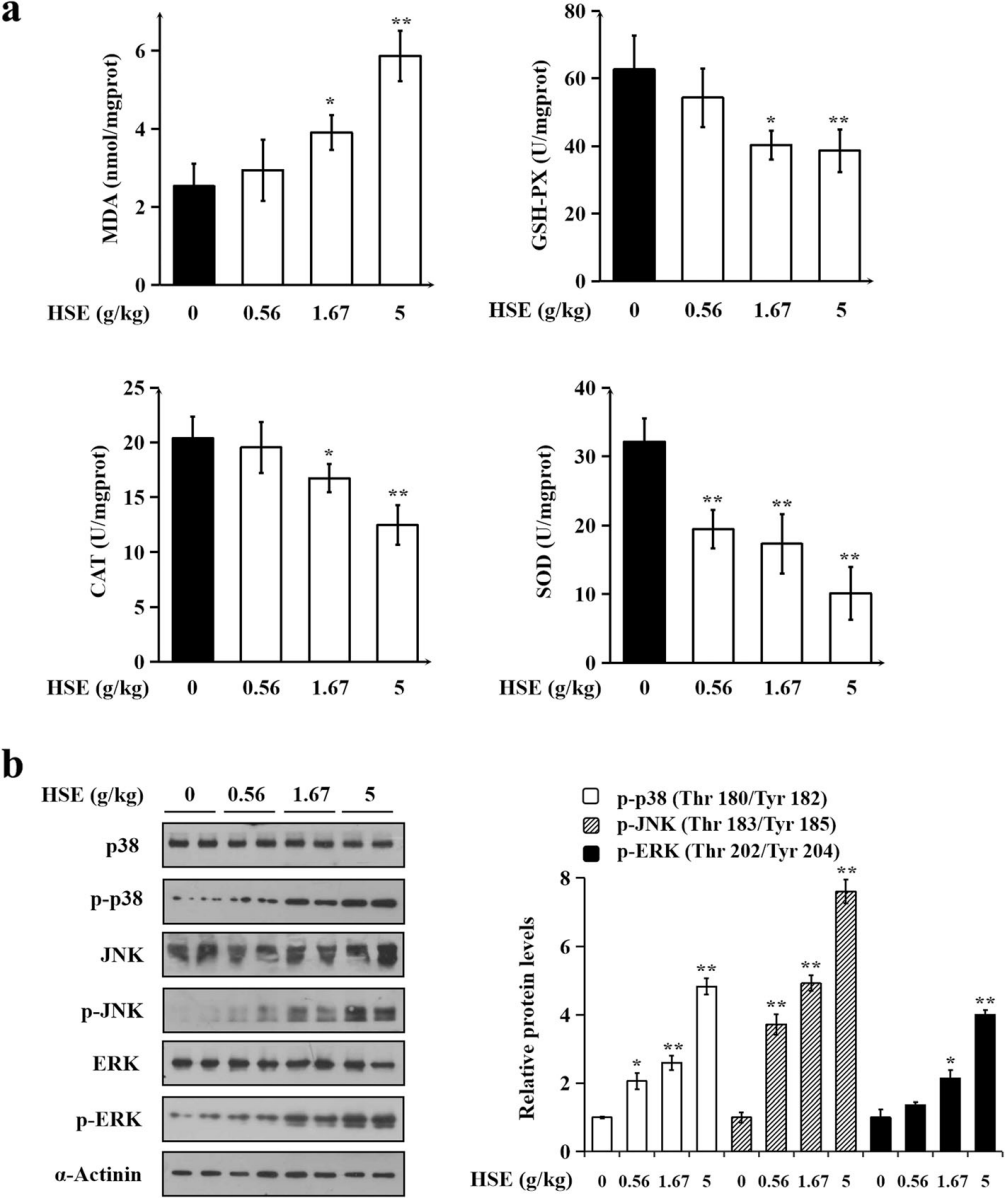
HSE caused oxidative stress, and induced the activation of MAPKs in lung tissues of rats. Animal treatments are the same as in Fig. 1. **a** Levels of MDA, and activities of GSH-PX, CAT and SOD in lung tissues. Values are expressed as mean ± SEM (*n* = 20). **b** Protein levels of p38, phospho-p38 (Thr180/Tyr182), JNK, phospho-JNK (Thr183/Tyr185), ERK and phospho-ERK (Thr202/Tyr204). α-Actinin served as a loading control. Representative immunoblotting results (left panel) and quantitative results (right panel) are shown. Data in bar charts are mean ± SEM of 3 independent experiments. In (**a**) and (**b**), * *P* < 0.05, ** *P* < 0.01 vs. control group

Treatment-induced oxidative stress is a mechanism of drug toxicity in tissues [20]. MDA, a product of lipid peroxidation, is the biomarker for estimating the status of oxidative stress [21]. As shown in Fig. 4a, MDA level in rat lung was significantly elevated after a 24-week i.g. treatment with 1.67 g/kg or 5 g/kg of HSE. In addition, activities of antioxidant enzymes GSH-PX, CAT and SOD in lung tissues were significantly decreased in 1.67 g/kg and 5 g/kg of HSE-treated groups compared to the control group. These results indicate that chronic treatment with HSE causes oxidative stress in lung tissues of rats.

Activation of MAPKs is reported to be responsible for the initiation and progression of drug-induced lung injury [22]. To investigate whether HSE affects MAPKs in lung tissues, we detected the protein levels of three MAPKs using immunoblotting. It was found that HSE treatment dose-dependently and significantly up-regulated protein levels of phospho-p38 (Thr180/Tyr182), phospho-ERK (Thr202/Tyr204) and phospho-JNK (Thr183/Tyr185) without affecting total p38, ERK and JNK (Fig. 4b), showing that HSE induced activation/phosphorylation of the three proteins in lung lysates. These results indicate that chronic treatment of HSE activated MAPKs in lung tissues.

Together, these findings indicate that a 24-week oral treatment with HSE causes decrease in body weight gain, hepatotoxicity and pulmonary toxicity in rats.

## Discussion

A sub-chronic toxicity study showed that aqueous extract of HS causes lung injury in mice [10]. In the present study, we found that 24-week oral dosing of HSE induces lung injury in rats. Drug-induced lung injury can be caused by oxidative stress and activation of MAPKs [23, 24]. Oxidative stress is associated with increased production of oxidizing species and decreased effectiveness of antioxidant defenses [20]. In animals, the lung is protected against oxidative damage by antioxidative enzymes, such as SOD, CAT and GSH-PX [25]. Results of the present study showed that HSE decreases the activity of antioxidant enzymes and increases the production of MDA, a product of lipid peroxidation, in rat lung tissues. These findings suggest that treatment-induced oxidative stress is one of the mechanisms responsible for the pulmonary toxicity caused by long-term dosing of HSE. Activation of MAPKs has also been linked to lung injury. MAPKs are a family of serine-threonine kinases that include ERK, JNK and p38, whose activation causes oxidative stress in the lung, leading to lung injury [26]. In this study, it was found that HSE dose-dependently induced activation of p38, ERK and JNK, indicating that activation of MAPKs is another mechanism underlying HSE-induced lung injury. Pro-inflammatory cytokines, such as IL-6, IL-1β and TNF-α, are also responsible for drug-induced lung injury [27]. Activation of MAPKs is reported to up-regulate the expression of pro-inflammatory cytokines in lung [28]. Unexpectedly, IL-6, IL-1β and TNF-α were not detectable (data not shown), although immune cell infiltration was observed, in lungs of HSE-treated rats. This may be because of negative regulations of the cytokines through other pathways affected by the multiple-component HSE, which needs to be tested in the future.

In the present study, we for the first time found that long-term treatment with HSE causes liver injury in rats. Elevated levels of inflammatory cytokines, such as IL-6, IL-1β and TNF-α, are commonly observed in experimental animals and patients with liver injury [29]. Over-production of these cytokines induces liver fibrosis and damage [30, 31]. In this study, it was found that hepatic levels of pro-inflammatory cytokines were up-regulated by HSE, indicating that inflammatory response contributes to HSE-induced hepatotoxicity. Total and phosphorylated MAPKs were examined in liver tissues as in lung tissues. Results showed that unlike in lung, HSE had no significant influence in MAPKs activation in liver (data not shown). Oxidative stress is one pathological mechanism of drug-induced liver injury [32]. In the present study, it was found that HSE did not affect the activity of antioxidant enzymes nor the production of MDA in rat liver tissues (data not shown), suggesting that HSE did not induce oxidative stress in rat liver. Why HSE caused inflammatory response but did not affect MAPKs activation and induce oxidative stress in livers of rats is a question to be addressed.

In the present study, we found that chronic dosing with HSE decreased rat body weight gain, while the food intake of rats was not significantly affected. Histopathological examinations showed that HSE did not cause histological changes in rat stomach and small intestine (data not shown). The HSE treatment-associated reduction in rat body weight gain may be due to its hepatotoxicity; this possibility needs to be confirmed.

There are limitations in the present study. For example, the stage of estrous cycle of female rats was not detected; and HSE’s NOAEL (no observed adverse effect level) could not be determined, because HSE at all three dose levels induced toxicities in rats. In the future, we will, on the basis of this study, conduct a standard assessment to establish the chronic toxicity profile of HS.

## Conclusions

In the present study, we for the first time demonstrated that long-term oral administration of HSE causes toxicities in rats evidenced by decreased body weight gain as well as liver and lung damage. Treatment-induced oxidative stress, inflammation, and MAPK activation are involved in HSE’s chronic toxicities. These potential consequences should be taken into account when using HS to treat chronic diseases.

## Supplementary Information

**Additional file 1: Figure S1.** An HPLC method developed for quality control of HSE. **(a)** HPLC chromatograms of the chemical marker kirenol (5.0 μg/mL) (upper panel) and HSE (lower panel). HPLC analysis was performed on an Agilent 1260 system equipped with a Diode-array detector. Separations were performed on an AlltimaTM C-18 analytical column (250 mm × 4.6 mm I.D., 5 μm) and an Alltima C-18 guard-column (12.5 mm × 4.6 mm I.D., 5 μm) maintained at 25 °C. Isocratic elution was performed with a mobile phase of A (0.1% phosphate acid solution, analytical grade, RCI Labscan Limited) and B (ACN, HPLC grade, RCI Labscan Limited) (70:30, v/v). The flow rate was maintained at 0.35 mL/min, and sample injection volume was 5 μL in each test. Since kirenol has a prominent absorption around 215 nm in the UV spectrum, 215 nm was chosen as the reference wavelength. **(b)** Contents of kirenol in HSE. **Figure S2**. Body weight changes of rats treated once with 5 g/kg of HSE. Rats were randomly divided into two groups: control group and 5 g/kg of HSE group, each 5 males and 5 females. Rats were i.g. administered with distilled water (control group) or 5 g/kg of HSE (5 g/kg of HSE group), and observed for 14 days. (a) Body weight of male rats. (b) Body weight of female rats. Values are expressed as mean ± SEM (*n* = 5). **Table S1**. Absolute and relative organ weights of rats in the 24-week oral dosing toxicity test. **Table S2**. Urinalyses of rats in the 24-week oral dosing toxicity test. **Table S3**. Hematological parameters of rats in the 24-week oral dosing toxicity test. **Table S4**. Serum assay parameters of rats in the 24-week oral dosing toxicity test.

**Additional file 2: Figure S3.** Original blot images of Western blot. Protein levels of p38, phospho-p38 (Thr180/Tyr182), JNK, phospho-JNK (Thr183/Tyr185), ERK and phospho-ERK (Thr202/Tyr204) and α-Actinin were shown. The bands were shown on different films because of different exposure time.

## Data Availability

The analyzed data supporting the conclusions of this article are included in this published article and its supplementary information files. The raw data are available from the corresponding author on reasonable request.

## Abbreviations

ALB: Albumin
ALP: Alkaline phosphatase
ALT: Alanine aminotransferase
AST: Aspartate aminotransferase
BUN: Blood urea nitrogen
Ca^2+^: Calcium
CAT: Catalase
CK: Creatine kinase
DAD: Diodearray detector
GLU: Blood glucose
GSH-PX: Phospholipid hydroperoxide glutathione peroxidase
HPLC: High-performance liquid chromatography
HS: Herba Siegesbeckiae
i.g.: intragastrically
IL: Interleukin
K^+^: Potassium
LD_50_: Median lethal dose
LDH: Lactate dehydrogenase
MAPK: Mitogen-activated protein kinase
MDA: Malonaldehyde
MTD: Maximum tolerated dose
Na^+^: Sodium
RA: Rheumatoid arthritis
SD: Sprague–Dawley
SOD: Superoxide dismutase
TBA: Total bile acid
TC: Total cholesterol
TCM: Traditional Chinese medicine
TNF: Tumor necrosis factor
TP: Total protein
