# Supplementary material for: Twenty-four-week oral dosing toxicities of Herba Siegesbeckiae in rats
Source: BMC Complement Med Ther. 2020 Nov 11;20:341. doi: 10.1186/s12906-020-03137-6 (PMC7661185; doi:10.1186/s12906-020-03137-6)
Supplement: Supplementary file 1 — Additional file 1: Figure S1. An HPLC method developed for quality control of HSE. (a) HPLC chromatograms of the chemical marker kirenol (5.0 μg/mL) (upper panel) and HSE (lower panel). HPLC analysis was performed on an Agilent 1260 system equipped with a Diode-array detector. Separations were performed on an AlltimaTM C-18 analytical column (250 mm × 4.6 mm I.D., 5 μm) and an Alltima C-18 guard-column (12.5 mm × 4.6 mm I.D., 5 μm) maintained at 25 °C. Isocratic elution was performed with a mobile phase of A (0.1% phosphate acid solution, analytical grade, RCI Labscan Limited) and B (ACN, HPLC grade, RCI Labscan Limited) (70:30, v/v). The flow rate was maintained at 0.35 mL/min, and sample injection volume was 5 μL in each test. Since kirenol has a prominent absorption around 215 nm in the UV spectrum, 215 nm was chosen as the reference wavelength. (b) Contents of kirenol in HSE. Figure S2. Body weight changes of rats treated once with 5 g/kg of HSE. Rats were randomly divided into two groups: control group and 5 g/kg of HSE group, each 5 males and 5 females. Rats were i.g. administered with distilled water (control group) or 5 g/kg of HSE (5 g/kg of HSE group), and observed for 14 days. (a) Body weight of male rats. (b) Body weight of female rats. Values are expressed as mean ± SEM (n = 5). Table S1. Absolute and relative organ weights of rats in the 24-week oral dosing toxicity test. Table S2. Urinalyses of rats in the 24-week oral dosing toxicity test. Table S3. Hematological parameters of rats in the 24-week oral dosing toxicity test. Table S4. Serum assay parameters of rats in the 24-week oral dosing toxicity test. [file 12906_2020_3137_MOESM1_ESM.docx]

**Additional file 1**


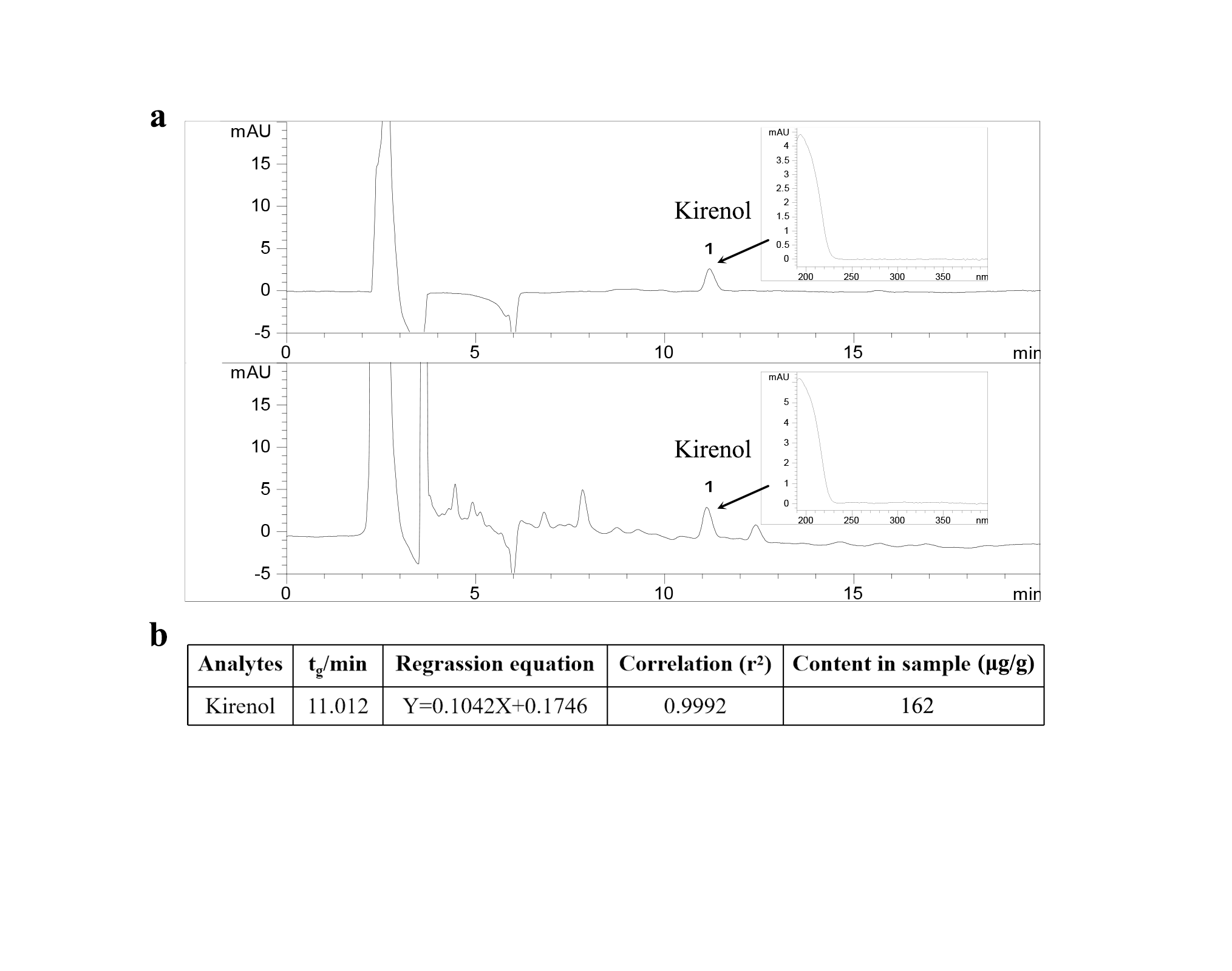


**Figure S1.** An HPLC method developed for quality control of HSE. **(a)** HPLC chromatograms of the chemical marker kirenol (5.0 µg/mL) (upper panel) and HSE (lower panel). HPLC analysis was performed on an Agilent 1260 system equipped with a Diode-array detector. Separations were performed on an Alltima^TM^ C-18 analytical column (250 mm × 4.6 mm I.D., 5 µm) and an Alltima C-18 guard-column (12.5 mm × 4.6 mm I.D., 5 µm) maintained at 25 °C. Isocratic elution was performed with a mobile phase of A (0.1% phosphate acid solution, analytical grade, RCI Labscan Limited) and B (ACN, HPLC grade, RCI Labscan Limited) (70:30, v/v). The flow rate was maintained at 0.35 mL/min, and sample injection volume was 5 μL in each test. Since kirenol has a prominent absorption around 215 nm in the UV spectrum, 215 nm was chosen as the reference wavelength. **(b)** Contents of kirenol in HSE.

**
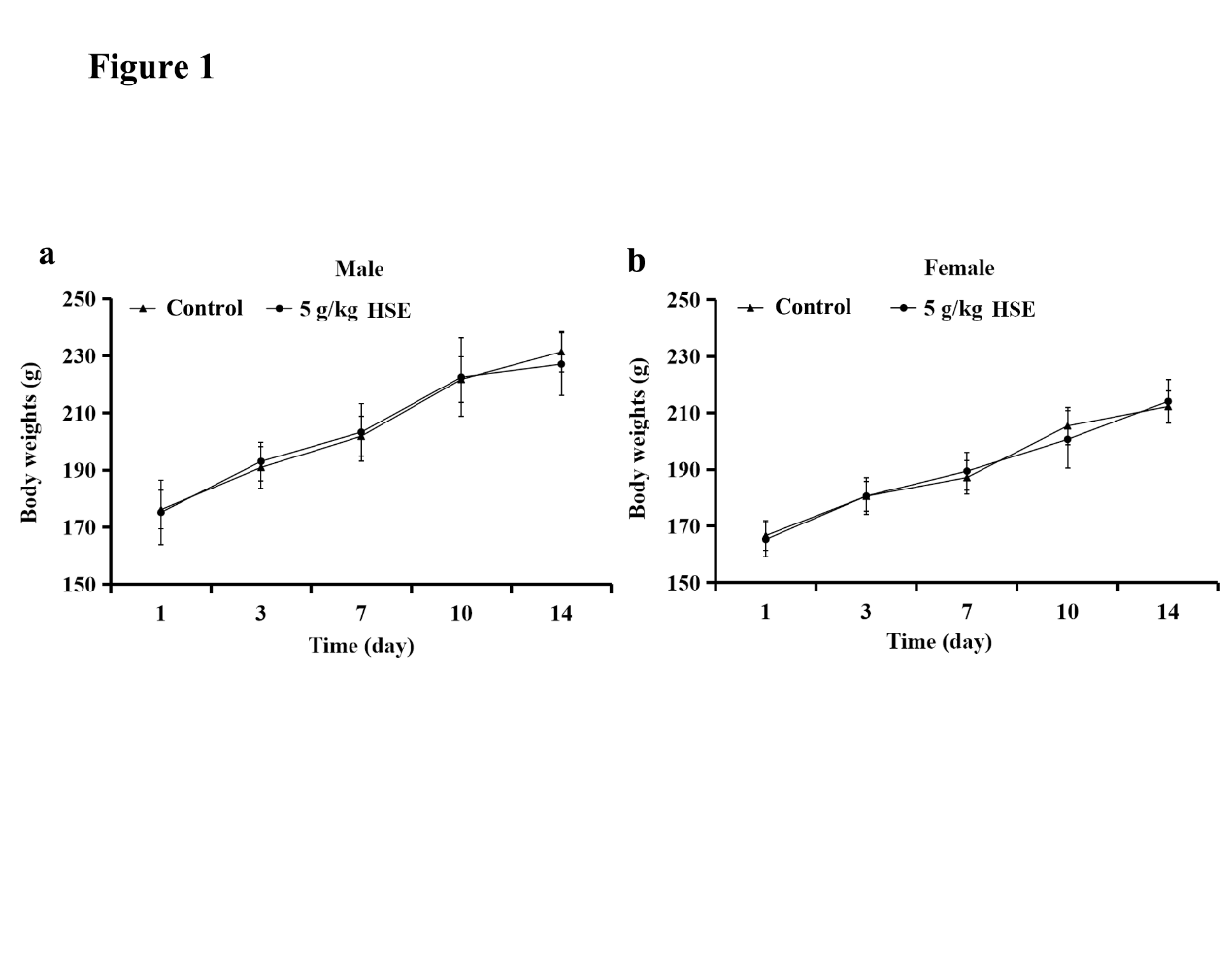
**

**Figure S2.** Body weight changes of rats treated once with 5 g/kg of HSE. Rats were randomly divided into two groups: control group and 5 g/kg of HSE group, each 5 males and 5 females. Rats were i.g. administered with distilled water (control group) or 5 g/kg of HSE (5 g/kg of HSE group), and observed for 14 days. **(a)** Body weight of male rats. **(b)** Body weight of female rats. Values are expressed as mean ± SEM (n = 5).

**Table S1** Absolute and relative organ weights of rats in the 24-week oral dosing toxicity test

|  | | | **Control** | **HSE** | | |
| --- | --- | --- | --- | --- | --- | --- |
|  |  |  |  | **5 g/kg** | **1.67 g/kg** | **0.56 g/kg** |
| Absolute organ weights | Heart (g) | ♂ | 1.33±0.02 | 1.24±0.07 | 1.22±0.09 | 1.22±0.09 |
|  |  | ♀ | 0.86±0.03 | 0.80±0.04 | 0.80±0.03 | 0.84±0.05 |
|  | Liver (g) | ♂ | 12.14±1.01 | 13.59±0.96 | 13.25±1.10 | 13.21±1.07 |
|  |  | ♀ | 6.92±0.30 | 7.77±0.23 | 7.31±0.56 | 7.12±0.33 |
|  | Spleen (g) | ♂ | 0.68±0.05 | 0.64±0.04 | 0.63±0.06 | 0.63±0.05 |
|  |  | ♀ | 0.38±0.02 | 0.34±0.03 | 0.36±0.02 | 0.36±0.03 |
|  | Lung (g) | ♂ | 1.53±0.11 | 1.65±0.17 | 1.62±0.06 | 1.62±0.09 |
|  |  | ♀ | 1.12±0.08 | 1.21±0.06 | 1.22±0.11 | 1.19±0.08 |
|  | Kidney (g) | ♂ | 2.92±0.17 | 2.71±0.23 | 2.73±0.15 | 2.72±0.15 |
|  |  | ♀ | 1.75±0.06 | 1.64±0.09 | 1.63±0.16 | 1.65±0.07 |
|  | Brain (g) | ♂ | 2.15±0.17 | 1.94±0.16 | 2.04±0.13 | 2.01±0.13 |
|  |  | ♀ | 1.85±0.04 | 1.73±0.09 | 1.74±0.08 | 1.74±0.10 |
|  | Adrenal Gland (mg) | ♂ | 96.40±10.81 | 93.20±7.69 | 92.40±9.21 | 91.60±9.21 |
|  |  | ♀ | 80.00±5.79 | 71.80±4.97 | 71.00±9.51 | 76.20±6.22 |
|  | Testis (g)  /Ovary (mg) | ♂ | 4.58±0.39 | 4.09±0.05 | 4.29±0.16 | 4.28±0.20 |
|  |  | ♀ | 48.20±4.60 | 48.40±3.91 | 48.40±3.58 | 50.80±5.07 |
|  | Epididymis (g)  /Uterus (mg) | ♂ | 0.43±0.02 | 0.39±0.03 | 0.41±0.01 | 0.40±0.02 |
|  |  | ♀ | 71.40±5.55 | 64.20±5.67 | 68.60±9.31 | 63.20±9.31 |
| Relative organ weights | Heart (%) | ♂ | 0.276±0.010 | 0.276±0.019 | 0.270±0.017 | 0.265±0.018 |
|  |  | ♀ | 0.261±0.012 | 0.265±0.022 | 0.257±0.014 | 0.267±0.011 |
|  | Liver (%) | ♂ | 2.525±0.168 | 3.023±0.192^*^ | 2.907±0.245^*^ | 2.868±0.233^*^ |
|  |  | ♀ | 2.104±0.137 | 2.559±0.170^*^ | 2.353±0.218^*^ | 2.270±0.146^*^ |
|  | Spleen (%) | ♂ | 0.142±0.010 | 0.142±0.011 | 0.138±0.012 | 0.136±0.010 |
|  |  | ♀ | 0.115±0.005 | 0.113±0.011 | 0.114±0.009 | 0.114±0.007 |
|  | Lung (%) | ♂ | 0.320±0.019 | 0.367±0.032^*^ | 0.357±0.013^*^ | 0.351±0.018^*^ |
|  |  | ♀ | 0.341±0.023 | 0.399±0.028^*^ | 0.392±0.044^*^ | 0.379±0.030^*^ |
|  | Kidney (%) | ♂ | 0.608±0.046 | 0.603±0.050 | 0.602±0.034 | 0.591±0.035 |
|  |  | ♀ | 0.533±0.018 | 0.539±0.042 | 0.525±0.061 | 0.526±0.030 |
|  | Brain (%) | ♂ | 0.448±0.035 | 0.430±0.031 | 0.449±0.031 | 0.437±0.030 |
|  |  | ♀ | 0.567±0.019 | 0.567±0.029 | 0.560±0.043 | 0.550±0.042 |
|  | Adrenal Gland (%) | ♂ | 0.020±0.002 | 0.021±0.002 | 0.020±0.002 | 0.020±0.002 |
|  |  | ♀ | 0.024±0.002 | 0.024±0.002 | 0.023±0.003 | 0.024±0.002 |
|  | Testis (%)  /Ovary (%) | ♂ | 0.954±0.094 | 0.911±0.017 | 0.945±0.045 | 0.929±0.051 |
|  |  | ♀ | 0.015±0.001 | 0.016±0.002 | 0.015±0.001 | 0.016±0.001 |
|  | Epididymis (%)  /Uterus (%) | ♂ | 0.089±0.005 | 0.087±0.008 | 0.090±0.004 | 0.086±0.004 |
|  |  | ♀ | 0.022±0.001 | 0.021±0.002 | 0.022±0.001 | 0.020±0.003 |

Values are mean ± SEM (n=10). ^*^*P*<0.05 *vs.* control group.

**Table S2** Urinalyses of rats in the 24-week oral dosing toxicity test

|  | | | **Control** | **HSE** | | |
| --- | --- | --- | --- | --- | --- | --- |
|  |  |  |  | **5 g/kg** | **1.67 g/kg** | **0.56 g/kg** |
| Vol (mL) | | ♂ | 14.6±1.1 | 15.0±0.7 | 14.8±0.8 | 14.6±0.9 |
|  |  | ♀ | 10.2±1.1 | 10.6±2.8 | 10.8±3.0 | 9.2±2.2 |
| Specific Gravity | | ♂ | 1.03±0.0 | 1.01±0.0 | 1.02±0.0 | 1.03±0.0 |
|  |  | ♀ | 1.02±0.0 | 1.02±0.0 | 1.03±0.0 | 1.02±0.0 |
| pH | | ♂ | 6.8±1.1 | 7.2±1.1 | 6.8±0.8 | 7.0±1.0 |
|  |  | ♀ | 7.2±0.8 | 7.2±0.8 | 6.6±0.9 | 7.2±1.3 |
| Leukocyte | Negative (n=) | ♂ | 8 | 9 | 8 | 8 |
|  |  | ♀ | 9 | 8 | 9 | 7 |
|  | Positive  (n=) | ♂ | 2 | 1 | 2 | 2 |
|  |  | ♀ | 1 | 2 | 1 | 3 |
| Nitrite | Negative (n=) | ♂ | 9 | 10 | 9 | 7 |
|  |  | ♀ | 8 | 9 | 8 | 8 |
|  | Positive  (n=) | ♂ | 1 | 0 | 1 | 3 |
|  |  | ♀ | 2 | 1 | 2 | 2 |
| Protein | Negative (n=) | ♂ | 7 | 8 | 9 | 8 |
|  |  | ♀ | 10 | 7 | 8 | 6 |
|  | Positive  (n=) | ♂ | 3 | 2 | 1 | 2 |
|  |  | ♀ | 0 | 3 | 2 | 4 |
| Glucose | Negative (n=) | ♂ | 10 | 9 | 6 | 7 |
|  |  | ♀ | 8 | 9 | 10 | 8 |
|  | Positive  (n=) | ♂ | 0 | 1 | 4 | 3 |
|  |  | ♀ | 2 | 1 | 0 | 2 |
| Ketone bodies | Negative (n=) | ♂ | 9 | 8 | 8 | 8 |
|  |  | ♀ | 9 | 8 | 9 | 10 |
|  | Positive  (n=) | ♂ | 1 | 2 | 2 | 2 |
|  |  | ♀ | 1 | 2 | 1 | 0 |
| Urobilino-gen | Negative (n=) | ♂ | 9 | 6 | 7 | 8 |
|  |  | ♀ | 10 | 6 | 6 | 7 |
|  | Positive  (n=) | ♂ | 1 | 4 | 3 | 2 |
|  |  | ♀ | 0 | 4 | 4 | 3 |
| Bilirubin | Negative (n=) | ♂ | 8 | 7 | 7 | 8 |
|  |  | ♀ | 9 | 6 | 7 | 7 |
|  | Positive  (n=) | ♂ | 2 | 3 | 3 | 2 |
|  |  | ♀ | 1 | 4 | 3 | 3 |
| Erythroc-yte | Negative (n=) | ♂ | 8 | 8 | 8 | 9 |
|  |  | ♀ | 8 | 8 | 9 | 8 |
|  | Positive  (n=) | ♂ | 2 | 2 | 2 | 1 |
|  |  | ♀ | 2 | 2 | 1 | 2 |

Values are mean ± SEM (n=10).

**Table S3** Hematological parameters of rats in the 24-week oral dosing toxicity test

|  | | | **Control** | **HSE** | | |
| --- | --- | --- | --- | --- | --- | --- |
|  |  |  |  | **5 g/kg** | **1.67 g/kg** | **0.56 g/kg** |
| Leukocyte (×10^9/L) | | ♂ | 10.00±0.44 | 11.68±0.95 | 11.32±0.74 | 11.54±0.86 |
|  |  | ♀ | 9.02±0.92 | 9.98±1.14 | 9.68±0.67 | 9.80±0.57 |
| Differential leukocyte count (%) | Neutrophil | ♂ | 17.95±0.82 | 19.46±0.62 | 18.90±1.24 | 19.18±1.34 |
|  |  | ♀ | 16.02±1.35 | 17.08±0.82 | 17.32±1.31 | 16.58±0.81 |
|  | Lymphocyte | ♂ | 77.46±0.76 | 77.06±1.07 | 77.16±1.52 | 76.08±1.55 |
|  |  | ♀ | 79.86±1.34 | 78.62±1.94 | 78.48±1.33 | 79.48±0.88 |
|  | Monocyte | ♂ | 2.09±0.07 | 1.88±0.44 | 2.22±0.36 | 2.86±1.04 |
|  |  | ♀ | 2.22±0.57 | 2.38±0.68 | 2.16±0.55 | 2.18±0.90 |
|  | Eosinophil | ♂ | 1.42±0.26 | 1.30±0.29 | 1.26±0.33 | 1.62±0.46 |
|  |  | ♀ | 1.52±0.31 | 1.54±0.68 | 1.70±0.36 | 1.54±0.35 |
|  | Basophil | ♂ | 0.20±0.00 | 0.30±0.14 | 0.46±0.19 | 0.26±0.15 |
|  |  | ♀ | 0.38±0.13 | 0.38±0.25 | 0.34±0.21 | 0.22±0.16 |
| Haemoglobin (g/dL) | | ♂ | 15.40±0.60 | 15.82±0.68 | 17.16±2.73 | 16.58±2.31 |
|  |  | ♀ | 15.43±0.23 | 15.46±0.67 | 14.88±1.00 | 15.52±1.54 |
| Haematocrit (L/L) | | ♂ | 0.48±0.01 | 0.48±0.01 | 0.47±0.02 | 0.46±0.05 |
|  |  | ♀ | 0.47±0.03 | 0.47±0.02 | 0.49±0.01 | 0.48±0.02 |
| Platelet Count (×10^9/L) | | ♂ | 910.20±59.31 | 890.40±24.58 | 870.60±50.24 | 914.00±60.35 |
|  |  | ♀ | 903.00±39.08 | 910.40±45.53 | 906.00±36.52 | 928.40±47.29 |
| Prothrombin Time (sec.) | | ♂ | 16.66±1.04 | 16.06±1.00 | 15.92±1.01 | 15.86±1.17 |
|  |  | ♀ | 15.62±1.23 | 15.80±0.94 | 15.38±1.88 | 14.10±0.87 |

Values are mean ± SEM (n=10). ^*^*P*<0.05 *vs.* control group.

**Table S4** Serum assay parameters of rats in the 24-week oral dosing toxicity test

|  | | **Control** | **HSE** | | |
| --- | --- | --- | --- | --- | --- |
|  |  |  | **5 g/kg** | **1.67 g/kg** | **0.56 g/kg** |
| Na^+^ (mmol/L) | ♂ | 148.61±13.89 | 148.41±15.50 | 143.00±18.93 | 153.94±14.98 |
|  | ♀ | 151.39±25.00 | 148.04±15.79 | 155.20±24.60 | 151.02±20.00 |
| K^+^ (mmol/L) | ♂ | 5.40±0.66 | 5.37±0.68 | 6.15±0.73 | 5.49±0.82 |
|  | ♀ | 4.45±0.35 | 4.43±0.26 | 4.62±0.66 | 4.36±0.15 |
| Ca^2+^ (mmol/L) | ♂ | 2.75±0.24 | 2.56±0.19 | 2.67±0.24 | 2.69±0.24 |
|  | ♀ | 2.68±0.19 | 2.71±0.31 | 2.68±0.31 | 2.64±0.29 |
| CK (U/L) | ♂ | 361.94±42.92 | 381.31±71.72 | 329.16±63.30 | 357.47±68.80 |
|  | ♀ | 373.86±66.71 | 402.16±46.40 | 381.31±65.03 | 357.47±21.46 |
| BUN (mmol/L) | ♂ | 5.70±0.71 | 6.42±1.17 | 5.63±0.67 | 5.56±0.66 |
|  | ♀ | 7.38±0.84 | 7.39±0.89 | 6.74±0.45 | 7.19±0.50 |
| CRE (μmol/L) | ♂ | 39.27±4.98 | 43.57±3.17 | 40.90±3.73 | 42.61±5.05 |
|  | ♀ | 38.68±5.24 | 41.66±4.85 | 41.29±6.18 | 39.49±3.11 |
| TP (g/L) | ♂ | 65.15±3.26 | 67.31±5.73 | 65.99±6.08 | 64.02±3.33 |
|  | ♀ | 66.41±2.53 | 70.66±4.97 | 66.53±5.79 | 65.87±5.48 |
| Albumin (g/L) | ♂ | 45.44±2.84 | 37.81±6.63 | 39.22±6.84 | 41.16±5.92 |
|  | ♀ | 40.76±4.60 | 36.21±4.00 | 39.95±4.53 | 42.36±4.06 |
| GLU (mmol/L) | ♂ | 126.36±16.12 | 131.04±16.02 | 127.08±14.77 | 128.52±14.32 |
|  | ♀ | 135.72±9.58 | 131.04±11.90 | 125.64±16.37 | 123.84±15.35 |
| TC (mmol/L) | ♂ | 1.70±0.13 | 1.48±0.16 | 1.46±0.21 | 1.53±0.32 |
|  | ♀ | 1.76±0.10 | 1.57±0.20 | 1.65±0.20 | 1.63±0.16 |
| TBA (μmol/L) | ♂ | 15.57±2.84 | 13.60±7.22 | 11.18±5.56 | 10.31±3.69 |
|  | ♀ | 10.53±4.21 | 14.98±3.61 | 13.77±3.25 | 13.16±7.37 |
| ALP (U/L) | ♂ | 77.03±11.28 | 133.53±22.93 | 120.46±19.91^*^ | 109.01±12.62^*^ |
|  | ♀ | 62.78±6.80 | 96.37±12.73^**^ | 80.08±8.89^*^ | 75.84±9.70^*^ |
| AST (U/L) | ♂ | 144.76±37.37 | 365.25±62.99^**^ | 347.94±75.39^**^ | 311.66±63.65^**^ |
|  | ♀ | 129.42±17.97 | 390.88±68.32^**^ | 409.82±50.59^**^ | 281.88±80.44^*^ |
| ALT (U/L) | ♂ | 77.10±10.50 | 280.22±64.12^**^ | 232.39±54.03^**^ | 184.49±41.22^**^ |
|  | ♀ | 52.62±8.21 | 299.35±50.19^**^ | 257.49±48.93^**^ | 156.38±36.16^*^ |
| LDH (U/L) | ♂ | 593.74±27.78 | 728.58±62.10^*^ | 707.58±62.10^*^ | 681.30±23.49^*^ |
|  | ♀ | 615.10±44.99 | 747.00±41.00^**^ | 723.30±42.09^*^ | 710.46±37.76^*^ |

Values are mean ± SEM (n=10). ^*^*P*<0.05, ^**^*P*<0.01 *vs.* control group.
