# Supplementary material for: Twenty-four-week oral dosing toxicities of Herba Siegesbeckiae in rats
Source: BMC Complement Med Ther. 2020 Nov 11;20:341. doi: 10.1186/s12906-020-03137-6 (PMC7661185; doi:10.1186/s12906-020-03137-6)
Supplement: Supplementary file 2 — Additional file 2: Figure S3. Original blot images of Western blot. Protein levels of p38, phospho-p38 (Thr180/Tyr182), JNK, phospho-JNK (Thr183/Tyr185), ERK and phospho-ERK (Thr202/Tyr204) and α-Actinin were shown. The bands were shown on different films because of different exposure time. [file 12906_2020_3137_MOESM2_ESM.doc]

**Additional file 2**


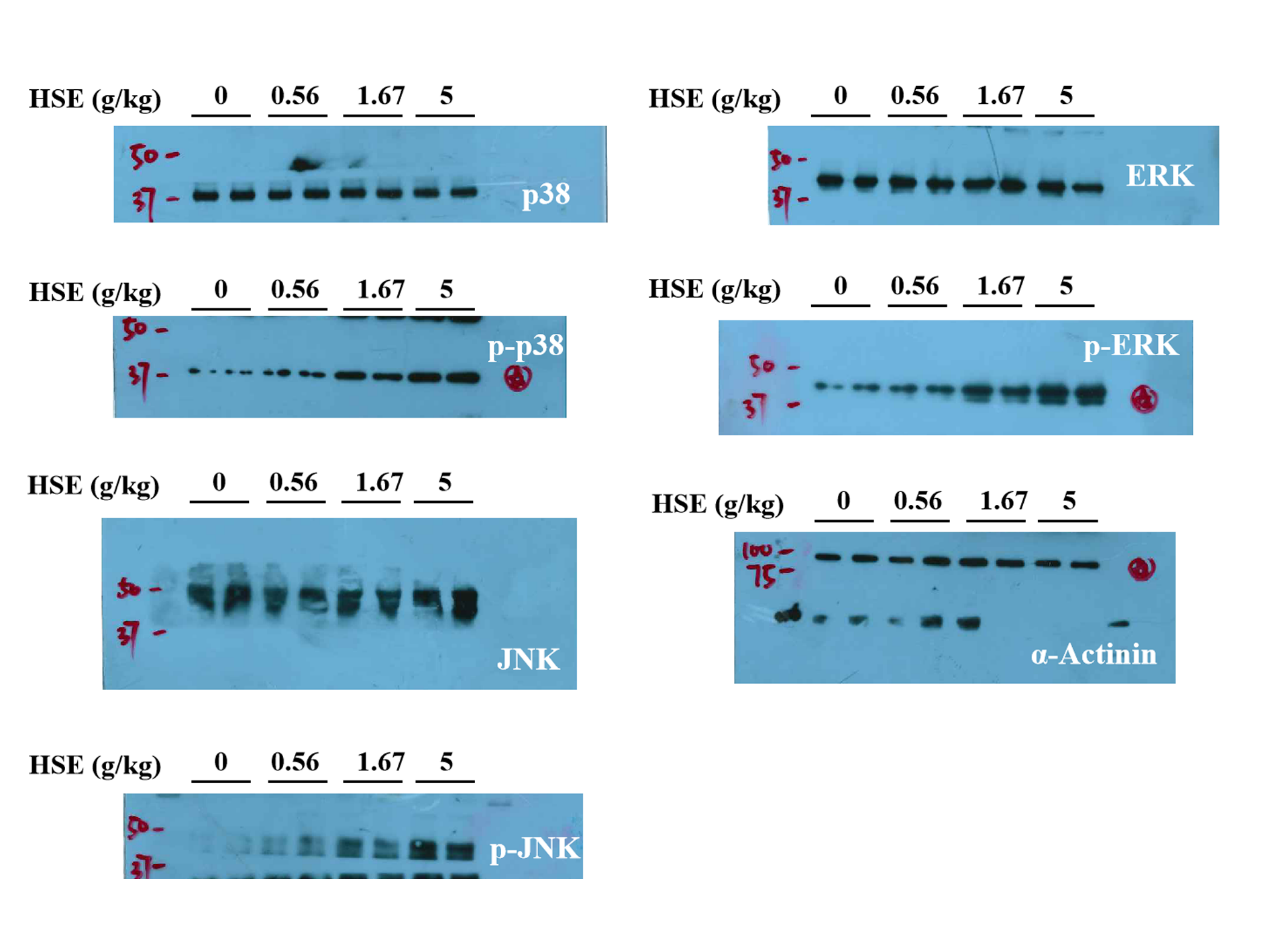


**Figure S3** Original blot images of Western blot. Protein levels of p38, phospho-p38 (Thr180/Tyr182), JNK, phospho-JNK (Thr183/Tyr185), ERK and phospho-ERK (Thr202/Tyr204) and α-Actinin were shown. The bands were shown on different films because of different exposure time.
